# Microbial foodborne outbreaks in Africa: a systematic review

**DOI:** 10.1093/inthealth/ihaf058

**Published:** 2025-06-24

**Authors:** Famous K Sosah, Eric S Donkor

**Affiliations:** Department of Medical Microbiology, University of Ghana Medical School, P.O. Box KB 4236, Korle Bu, Accra, Ghana; Department of Medical Microbiology, University of Ghana Medical School, P.O. Box KB 4236, Korle Bu, Accra, Ghana

**Keywords:** Africa, food contamination, food safety, foodborne illness, foodborne pathogens, hygiene, microbial foodborne outbreaks, public health

## Abstract

Microbial foodborne outbreaks are a severe public health challenge in Africa, which bears the highest global burden due to systemic vulnerabilities. Common microbial pathogens contaminate various foods, particularly raw and processed meats, leading to significant morbidity, mortality and economic losses. In this review, data from 31 studies encompassing 42 microbial foodborne outbreaks in Africa were synthesized, analyzed and visualized. Overall, 877 067 of infections and intoxications occurred, with 2064 hospitalizations and 2061 deaths. *Salmonella enterica* accounted for the most of infections and intoxication (53.6%), while *Staphylococcus aureus* caused the highest rate of outbreaks (21.4%). Clostridium botulinum was associated with the highest fatality (46.154%), followed by Listeria monocytogenes (20.323%). The implicated food vehicles in the outbreaks included processed meats (38.1%), cereals, legumes and tuber (21.43%), vegetables (9.52%) and seafood (7.14%). The key contributing factors were poor hygiene, inadequate food storage and limited compliance with food safety practices. Addressing critical deficiencies in food safety infrastructure, public awareness and hygiene practices requires enhanced surveillance systems, stricter regulatory frameworks, investment in infrastructure and public education campaigns. Efforts should prioritize the control of prevalent pathogens to mitigate the health and socioeconomic impacts of foodborne illnesses across the continent.

## Introduction

Foodborne outbreaks are a pervasive global public health issue, affecting an estimated 600 million people and causing 420 000 deaths annually, according to the WHO.^1–3^ Alarmingly, the burden of foodborne outbreak is not evenly distributed, with low- and middle-income countries shouldering most cases. Africa bears the highest per capita burden of foodborne outbreaks globally, driven by a combination of systemic vulnerabilities, including limited food safety infrastructure and regulatory enforcement.^2,3^ In 2010, the global burden of foodborne outbreak was estimated at 33 million disability-adjusted life years, with children aged <5 y accounting for 40% of this burden.^3^ These staggering figures highlight the urgent need to address food safety challenges across the continent.

Microbial pathogens are the leading cause of foodborne outbreaks in Africa, and are commonly found in both raw and ready-to-eat foods. Pathogens such as *Salmonella, Escherichia coli, Staphylococcus aureus* and *Listeria monocytogenes* have been identified as major contributors to foodborne illnesses, often contaminating animal products, fresh produce and water.^4–7^ The high prevalence of microbial contamination is exacerbated by inadequate food safety practices, poor sanitation and limited access to clean water, making outbreaks both frequent and severe.^8^ These outbreaks result not only in significant morbidity and mortality but also in substantial economic losses, further straining already fragile healthcare systems in Africa.^9,10^

Despite the significant public health implications, foodborne diseases caused by pathogens in Africa have not received the same level of attention as other major infectious diseases such as HIV/AIDS, malaria and TB, despite having a comparable burden.^2,3^ Furthermore, there remains a lack of comprehensive data on the epidemiology, causative agents and underlying drivers of microbial foodborne outbreaks on the continent. This lack of comprehensive data hinders the development of effective interventions and policies to mitigate the issue. Therefore, this study aims to review the existing evidence on microbial foodborne outbreaks in Africa, focusing on the magnitude of the outbreaks, distribution, most implicated food products and most prevalent foodborne pathogens, while proffering solutions to combat future outbreaks.

## Methods

### Data sources and search strategy

This systematic review was conducted following the Preferred Reporting Items for Systematic Reviews and Meta-Analysis (PRISMA) guidelines.^11^ The PRISMA guidelines offer a detailed checklist and flow diagram that assist in record identification, screening and evaluation. The literature search was conducted during 5–7 October 2024 across electronic databases, including Scopus, Web of Science, Google Scholar and PubMed, to identify articles related to microbial foodborne outbreak in Africa with no restrictions in years. Search terms included both Medical Subject Headings (MeSH terms) and keywords such as (‘Foodborne Diseases’[Mesh] OR ‘foodborne outbreak*’ OR ‘foodborne illness*’ OR ‘foodborne infection*’ OR ‘food poisoning’ OR ‘food contamination’) AND (‘Infections’[Mesh] OR ‘microbial infection*’ OR ‘bacterial infection*’ OR ‘viral infection*’ OR ‘parasitic infection*’ OR ‘fungal infection*’ OR ‘pathogen*’) AND (‘Africa’[Mesh] OR ‘Africa’ OR ‘Sub-Saharan Africa’ OR ‘North Africa’ OR ‘West Africa’ OR ‘East Africa’ OR ‘Central Africa’ OR ‘Southern Africa’). The reference lists of the identified articles were carefully examined to include all relevant studies. The full search strategy of the various electronic databases used is shown in Supplementary Table 1.

### Inclusion criteria

The review carefully examined studies conducted across all years with evidence of microbial foodborne outbreaks in Africa. For this study, a microbial foodborne outbreak occurs when two or more individuals experience a similar illness due to ingesting a typical food containing pathogens or toxins. Hence, a single case study was excluded from this review. Peer-reviewed articles published in English and other languages were included. Studies in other languages apart from English were translated using Google Translate (Manufactured by Google LLC – Mountain View, California). All study design types were included, while studies not providing information on five or more of the following inclusion criteria were excluded:

1. number of people infected by the microbial outbreak
2. identification of implicated food (food vehicle)
3. outbreak cases were laboratory confirmed
4. number of reported deaths
5. identification of pathogen involved in the outbreak
6. number of hospitalizations
7. the country where the outbreak occurred
8. year of outbreak occurrence.

### Study selection

The selection of studies for this review involved a three-phased screening process to retrieve relevant articles. Duplicates were first removed from the initial pool of studies using the Rayyan tool for systematic reviews. The titles and abstracts of the remaining studies were then screened to evaluate their alignment with the predefined inclusion criteria. In the final stage, the full texts of the studies that passed the initial screening were thoroughly examined against the established inclusion and exclusion criteria to determine their eligibility for inclusion in the review.

**Figure 1.**
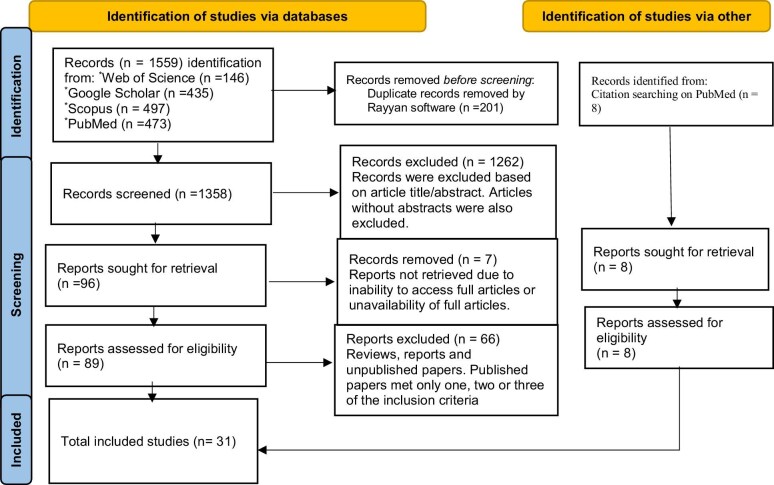
PRISMA flow diagram for the identification, screening and evaluation of the articles included in the study. PRISMA: Preferred Reporting Items for Systematic Reviews and Meta-Analysis.

Only the full-text studies that provided original data on microbial foodborne outbreaks in Africa and met the inclusion criteria were ultimately selected for inclusion in the review. The included articles were then downloaded using the Zotero reference management tool (version 7.0.3) to facilitate the review process. The article selection process is outlined by the PRISMA flow diagram in Figure 1.

### Data extraction and analysis

Microsoft Excel 365 software was used to manage the data from the studies reviewed. The data were independently extracted from individual studies via the data abstraction format prepared in Microsoft Excel 365. The extracted information included the author's name, the implicated microbial pathogens, the countries where the outbreaks occurred, the food vehicles, the number of people affected by the outbreak, laboratory confirmation of the outbreaks, the number of hospitalizations, the number of deaths and the year the outbreaks occurred. Tables, graphs and charts were used to visualize the distribution of the study characteristics and findings while geographical distribution of the included articles was visualized using a map.

## Results

### Search results

The initial search of the online databases identified a total of 1559 publications from various databases, including Web of Science (n=146), Google Scholar (n=435), Scopus (n=497), PubMed (n=473), and eight studies were identified through citation searching on PubMed. After removing duplicates (n=201), the titles and abstracts of the remaining 1358 records were screened. Among these, 1262 articles were excluded because they did not meet the established inclusion criteria for an outbreak. A total of 96 full-text articles were subsequently assessed for eligibility, of which 31^12–42^ met the inclusion criteria for the review (Figure 1).

### Characteristics of included studies

**Figure 2.**
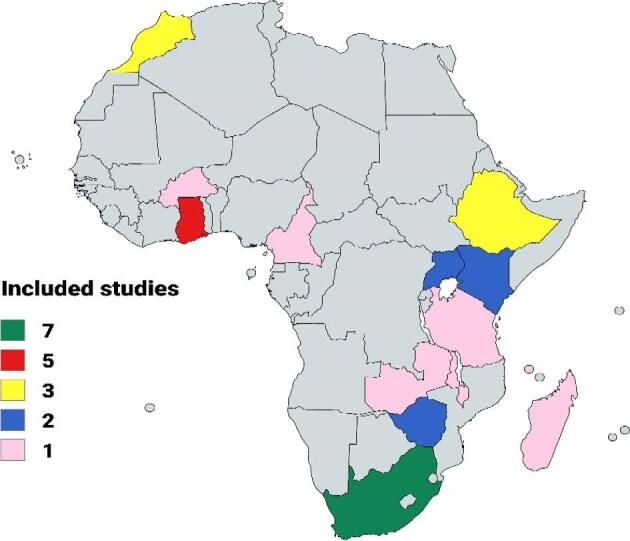
Geographical distribution of the included studies.

Table 1 shows the summary of the 31 reported microbial foodborne outbreaks in Africa included in this review. Geographically, South Africa recorded the highest number of microbial foodborne outbreaks (7; 22.58%), followed by Ghana with 5 (16.13%), Ethiopia and Morocco each with 3 (9.68%), Kenya, Uganda and Zimbabwe each with 2 (6.45%), while Burkina Faso, Cameroon, Comoros, Madagascar, Malawi, Tanzania and Zambia had 1 (3.23%) each. The geographical distribution of these included studies across Africa is shown in Figure 2.

**Table 1.** Foodborne outbreaks caused by microbial pathogens in Africa

									
Foodborne pathogen	Food vehicle	Main reason	No. of cases	Laboratory confirmed	Hospitalization	No. of deaths	Country	Year(s) of outbreak	References
									
									
*Campylobacter* spp.	Poultry meat	Not applicable	400 000	Not applicable	Not applicable	600	Burkina Faso	2017	Havelaar et al.^12^
*Clostridium botulinum* type A	Kochi-kocha, cheese and clarified butter	Ingestion of botulinum toxins in kochi-kocha, cheese and butter	10	10	3	5	Ethiopia	2015	Bacha et al.^13^
*Clostridium botulinum* type A	Oil-based condiment	Not applicable	3	3	3	1	Uganda	2008	Viray et al.^14^
*Clostridium perfrigens*	Stew and water	Contamination	68	Not applicable	Not applicable	0	Ghana	2015	Ameme et al.^28^
*Clostridium perfrigens*	Not applicable	Food left in temperature danger zone	10	10	Not applicable	3	South Africa	2013	Bamford et al.^15^
Cyanobacteria (toxins)	Shellfish	Contamination	75	26	61	0	Tanzania	2015	Urio et al.^16^
Cyanobacteria (toxins)	Sea turtle	A biotoxin ingested by the turtle	49	Not applicable	2	1	Comoros	2012	Ben et al.^17^
Cyanobacteria (toxins)	Sea turtle	Consumption of turtle	76	Not applicable	76	8	Madagascar	2014	Rasamimanana et al.^18^
*Escherichia coli O157*	Briouat	Contamination	50	Not applicable	0	0	Morocco	2017	Abdou et al.^19^
*Escherichia coli*	Raw meat and raw milk	Eating raw meat and raw milk	35	7	35	0	Ethiopia	2018	Kassahun and Wongiel^20^
*Listeria monocytogenes*	Ready-to-eat processed meat	Contamination	1060	1060	Not applicable	216	South Africa	2017/2018	Tchatchouang et al.^21^
*Listeria monocytogenes*	Polony (processed deli meat)	Not applicable	1034	1034	544	204	South Africa	2017	Olanya et al.^22^
*Listeria monocytogenes*	Polony (processed meat)	Contamination	937	567	2	196	South Africa	2017/2018	Thomas et al.^23^
Non-typhoidal *Salmonella enterica*	Vegetables	Not applicable	60 000	Not applicable	Not applicable	160	Burkina Faso	2017	Havelaar et al.^12^
*Plesiomonas shigelloides*	Fish, mayonnaise and boiled eggs with mayonnaise	Cross-contamination	24	17	2	0	Cameroon	2003	Wouafo et al.^24^
*Providencia alcalifaciens*	Mashed potatoes	Poor hygiene, inadequate food storage	11	11	4	0	Kenya	2013	Shah et al.^25^
*Salmonella enterica*	Poultry meat	Not applicable	400 000	Not applicable	Not applicable	600	Burkina Faso	2011	Havelaar et al.^12^
*Salmonella enterica*	Samp (processed maize meal)	The samp was improperly stored and prepared	164	164	9	1	South Africa	2018	Motladiile et al.^26^
*Salmonella enterica*	Beverage (juice) and water	Contamination	10 230	51	Not applicable	Not applicable	Uganda	2015	Kabwama et al.^27^
*Salmonella* spp.	Stew and water	Contamination	68	68	Not applicable	0	Ghana	2015	Ameme et al.^28^
*Salmonella* spp.	Eating raw meat and drinking raw milk	Eating raw meat and raw milk	35	7	35	0	Ethiopia	2018	Kassahun and Wongiel^20^
									
*Salmonella enteritidis*	Chicken meat	Not applicable	196	196	Not applicable	Note applicable	South Africa	2011–2012	Smith et al.^29^
*Salmonella enteritidis*	Goat liver	Dead goat from an illness	2	2	Not applicable	0	South Africa	2013	Muvhali et al.^30^
*Salmonella enteritidis*	Not applicable	Not applicable	46	Not applicable	6	0	South Africa	2014	Muvhali et al.^30^
*Salmonella enteritidis*	Not applicable	Not applicable	3	Not applicable	Not applicable	0	South Africa	2013	Muvhali et al.^30^
*Salmonella enteritidis*	Not applicable	Not applicable	65	Not applicable	8	Not applicable	South Africa	2014	Muvhali et al.^30^
*Salmonella enteritidis*	Not applicable	Not applicable	80	Not applicable	6	Not applicable	South Africa	2014	Muvhali et al.^30^
*Salmonella enteritidis*	Not applicable	Not applicable	10	Not applicable	Not applicable	Not applicable	South Africa	2014	Muvhali et al.^30^
*Salmonella enteritidis*	Chicken feet	Not applicable	4	4	Not applicable	0	South Africa	2015	Muvhali et al.^30^
*Staphylococcus aureus*	Vegetable salad	Not applicable	16	Not applicable	0	Not applicable	Ghana	2018	Adjei et al.^31^
*Staphylococcus aureus*	Rice, chicken and minced meat	Contamination	3	Not applicable	Not applicable	0	Morocco	2017	Abdou et al.^19^
*Staphylococcus aureus*	Fish, fries and soda	Non-compliance with hygiene practices	9	Not applicable	0	0	Morocco	2019	Elkhal et al.^32^
*Staphylococcus aureus*	Soup, chicken, salad and bread, with rice	Non-compliance with hygiene practices	6	Not applicable	0	0	Morocco	2022	Elkhal et al.^32^
*Staphylococcus aureus*	Chicken tagine	Improper thawing and storage of the chicken	9	Not applicable	Not applicable	Not applicable	Morocco	2017	Essayagh et al.^33^
*Staphylococcus aureus*	Cooked beans	Food contaminated by food handlers during preparation	59	Not applicable	59	0	Zambia	2017	Kapaya et al.^34^
*Staphylococcus aureus*	Stewed chicken	Cross-contamination	53	Not applicable	Not applicable	Not applicable	Zimbabwe	2014	Gumbo et al.^35^
*Staphylococcus aureus*	Chicken	Cross-contamination	40	Not applicable	Not applicable	0	Zimbabwe	2016	Sithole et al.^36^
*Staphylococcus aureus*	Briouat	Contamination	50	Not applicable	0	0	Morocco	2017	Abdou et al.^19^
*Vibrio cholerae*	Leftover cooked peas	Not heating the cooked peas	1931	8	1931	68	Malawi	1990	Swerdlow et al.^37^
*Vibrio cholerae*	Raw vegetables	Contamination	25	7	25	0	Ethiopia	2017	Dinede et al.^38^
*Vibrio cholerae*	Fufu and groundnut soup	Contamination	17	8	Not applicable	1	Ghana	2011	Acquah et al.^39^
*Vibrio cholerae*	Cooked chicken	Chicken was likely contaminated during packaging	249	24	Not applicable	Not applicable	Kenya	2017	Mwenda et al.^40^
Unidentified	Waakye and shito	Contamination	43	Not applicable	0	0	Ghana	2014	Ameme et al.^41^
Unidentified	Rice and groundnut soup	Not applicable	212	212	Not applicable	0	Ghana	2007	Malm et al.^42^

A total of 42 microbial foodborne outbreaks associated with various food products (Table 1) were recorded, with 877 067 individual cases, 2064 hospitalizations and 2061 deaths. Overall, 3496 of the cases were laboratory confirmed. A wide variety of microbial pathogens were implicated in foodborne outbreaks, including *Campylobacter* spp*., Clostridium botulinum, Clostridium perfringens, E. coli, L. monocytogenes, Salmonella* spp*., S. aureus, Vibrio cholerae, Plesiomonas shigelloides, Providencia alcalifaciens* and Cyanobacteria (toxins). Almost all the microbial pathogens were associated with specific food vehicles and varying outbreak scales, indicating the diverse etiology of foodborne diseases in Africa. The implicated food vehicles were diverse, reflecting regional dietary practices and food preparation methods. Commonly reported food items included processed and raw meat (poultry meat, deli meat, sausages, chicken, goat liver, raw meat), fish, vegetables, cereals, legumes, tuber and traditional Africa dishes (waakye, fufu, shito, kochi-kocha). Contamination sources included improper food handling, inadequate storage, cross-contamination and consumption of raw or undercooked foods. Poor hygiene practices, non-compliance with food safety guidelines and environmental contamination were frequently implicated as major contributors to the microbial foodborne outbreaks. Some of the outbreaks in the study, however, lacked detailed epidemiological investigation, with laboratory confirmation rates varying significantly across studies.

All the 31 studies included in this review only reported bacterial foodborne outbreaks. Some studies reported multiple microbial foodborne outbreaks, resulting in a combined total that exceeded the total number of individual studies included in this review. Although two studies did not identify the microbe(s) responsible for the foodborne outbreaks, they were included in this review because they met the inclusion criteria set for the study, and the signs and symptoms displayed by the people infected in those two studies suggest that they were the result of a microbial foodborne outbreak.

### Frequency of microbial foodborne outbreaks

**Figure 3.**
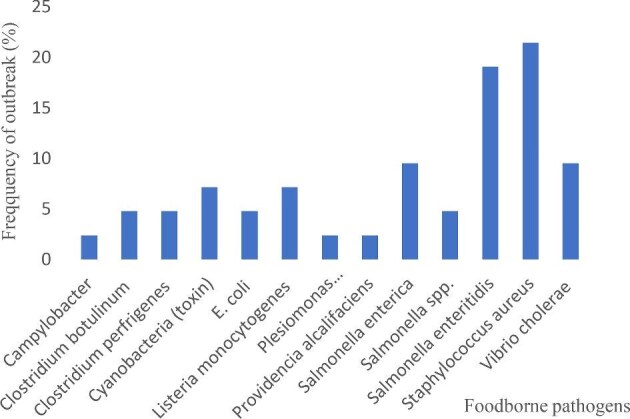
Frequency of microbial foodborne outbreaks in Africa.

Out of the 42 outbreaks, *S. aureus* had the highest frequency (9; 21.43%), followed by *Salmonella enteritidis* (8; 19.05%), then *Salmonella enterica* and *Vibrio cholerae* with 4 (9.52%) outbreaks each. *L. monocytogenes* and Cyanobacteria toxins were responsible for 3 (7.14%) outbreaks. *Salmonella* spp., *E. coli, C. botulinum* and *C. perfringens* caused 2 (4.77%) of the outbreaks each. The least number of microbial foodborne outbreaks (1; 2.38%) were caused by *Plesiomonas shigelloides, Providencia alcalifaciens* and *Campylobacter* spp. The frequency of microbial foodborne outbreaks is presented in Figure 3.

### The number of infected cases in outbreaks

The highest number of infected individual cases by microbial foodborne outbreaks were caused by *S. enterica* (470 394; 53.633%) (Table 1). Of these cases, 761 (0.162%) resulted in deaths; 215 (0.046%) of the cases were laboratory confirmed and 9 (0.002%) individuals were hospitalized. Also, 400 000 (45.61%) of the total individual cases were caused by *Campylobacter* spp.; this was the second highest number of recorded cases in this study with 600 (0.150%) deaths. *V. cholerae* was identified as causing 2222 (0.253%) cases with the highest number of hospitalized individuals (1956; 88.029%), resulting in 69 (3.105%) deaths. *C. botulinum* had the highest case fatality rate of 46.154% (6); out of 13 (0.001%) infected individual cases, 6 (46.154%) were hospitalized and all 13 (100%) were laboratory confirmed. *E. coli, P. shigelloides, P. alcalifaciens, Salmonella* spp*., S. enteritidis* and *S. aureus* were identified as causing individual cases ranging from 11 (0.001%) to 406 (0.046%), which resulted in no deaths. Among the 3031 (0.346%) cases caused by L. monocytogenes, 2661 (87.793%) were laboratory confirmed, which resulted in 616 (20.323%) deaths, the second highest death rate.

### Yearly outbreaks

**Figure 4.**
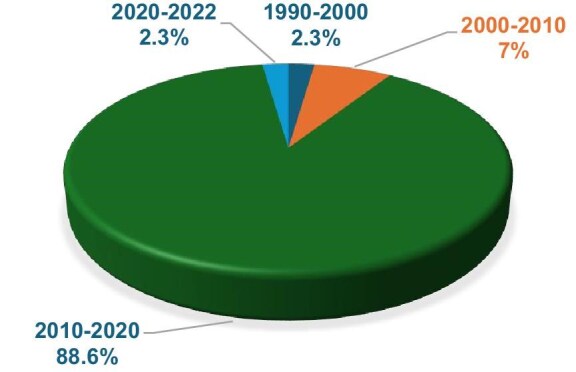
Occurrence of microbial foodborne outbreaks in Africa from 1990 to 2022.

The frequency of outbreaks (Figure 4) showed a significant increase over the decades, highlighting a growing burden of microbial foodborne outbreaks. From 1990 to 2000, only one (2.3%) outbreak was reported, while the period from 2000 to 2010 saw a modest rise with the occurrence of 3 (7%) outbreaks. However, the number of outbreaks surged dramatically from 2010 to 2020, with 39 microbial foodborne outbreaks reported during this decade alone, accounting for 88.6% of all recorded outbreak incidents. By contrast, only 1 (2.3%) outbreak occurred from 2020 to 2022, although this could reflect limited data availability or under-reporting for this more recent period.

### Food as vehicle for microbial foodborne outbreaks

**Figure 5.**
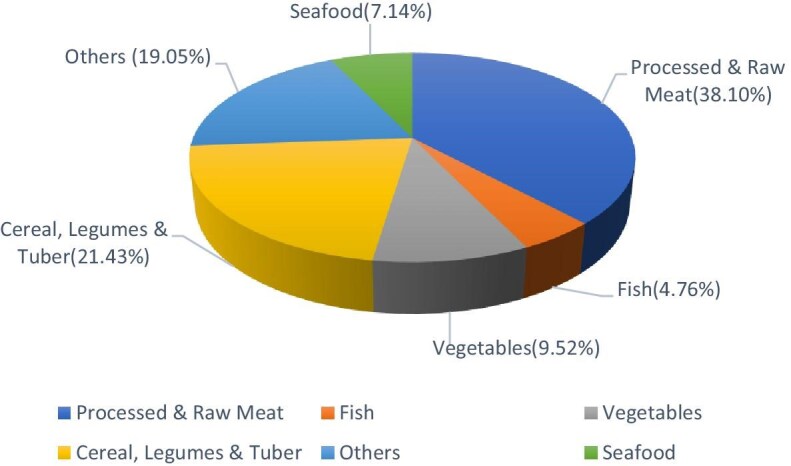
Microbial foodborne outbreaks related to food vehicles.

Figure 5 shows the analysis of implicated food vehicles associated with microbial foodborne outbreaks in Africa, revealing a diverse distribution of food categories contributing to these outbreak incidents. Processed and raw meat emerged as the most frequently implicated category, accounting for 16 (38.10%) of the reported microbial foodborne outbreaks. Cereal-based products, legumes and tuber were next, contributing to 9 (21.43%) outbreaks. Vegetables were implicated in 4 (9.52%) outbreaks, while seafood and fish accounted for 3 (7.14%) and 2 (4.76%), respectively. The remaining 8 (19.05%) outbreaks were linked to diverse traditional African dishes food categories.

## Discussion

The increasing prevalence of microbial foodborne outbreaks in Africa presents a significant public health challenge, exacerbated by multiple systemic factors. Poor sanitation, inadequate food safety practices and insufficient surveillance data on foodborne incidents are key contributors to this growing burden.^43^ Despite the implementation of government programs aimed at reducing outbreaks, the lack of financial investment, limited technical capacity and insufficient public awareness campaigns continue to hinder progress in mitigating these outbreaks.^44^

The major cause of microbial foodborne outbreaks in Africa is attributed to pathogenic bacteria, particularly *Salmonella* spp*., E. coli, S. aureus* and *L. monocytogenes*, which are commonly found in raw, ready-to-eat and animal-based foods.^5,45^ Among these pathogens, *Salmonella* spp. stands out as the most prevalent across the continent, with an average prevalence rate of 19.9% in raw foods and 21.7% in ready-to-eat foods.^5^ Alarmingly, studies in South Africa have identified even higher prevalence rates, with *Salmonella* serovars detected in 56.5% of animal (poultry) samples.^46^ The findings of these studies are similar to our findings in this review, that *Salmonella* spp., particularly *S. enterica*, is one of the leading causes of microbial foodborne outbreak cases with the highest number of infections (470 394; 53.633%) across Africa. For instance, in Burkina Faso and Uganda, outbreaks associated with *S. enterica* contamination resulted in approximately 470 230 individual cases from 2011 to 2017. These outbreaks were linked to a variety of food vehicles, including poultry meat, processed maize meal and beverages, demonstrating the pervasive nature of *S. enterica* contamination across diverse food systems. Similarly, in South Africa, *S. enterica* has been identified as a prominent pathogen responsible for outbreaks in both human and animal samples. The high number of individual cases linked to *S. enterica* reflects significant gaps in food handling, storage and preparation practices, as well as challenges in maintaining effective cold chain systems.^12^ These deficiencies are particularly pronounced in low- and middle-income countries, where infrastructure limitations exacerbate the risks of microbial contamination.^12^ In addition to *Salmonella, S. aureus* has also been identified as one of the largest contributors to microbial foodborne outbreaks in Africa, accounting for 21.43% of the reported occurrences of microbial foodborne outbreaks in this review. Epidemiological studies have shown a high prevalence of *S. aureus* contamination in both raw and ready-to-eat foods across Africa. On average, contamination rates of 27.8% in raw foods and 25.1% in ready-to-eat foods have been reported.^5^ This is in accordance with chicken, fish, bread, rice, fries and soda as the food products implicated by *S. aureus* in this study. The pathogen's ability to produce heat-stable toxins allows it to persist in food even after cooking, posing a unique challenge to food safety efforts.^47,48^ This could explain why 56 and 53 individuals were infected with *S. aureus* in cooked beans and stewed chicken, respectively. Unlike many other pathogens that can be eradicated through heat treatment, the resilience of *S. aureus* toxins underscores the importance of strict hygiene practices along the entire food production and handling chain.^49^

Besides the frequency of, and the number of infected individuals affected by the outbreaks in the current study, there is a high fatality rate associated with *L. monocytogenes* and *C. botulinum* microbial pathogens. In South Africa, the incidence of listeriosis caused by *L. monocytogenes* linked to ready-to-eat processed meats, which occurred during 2017–2018, claimed >200 lives with each outbreak reported, making it one of the deadliest foodborne outbreaks ever recorded globally. Despite being relatively rare, listeriosis ranks second after salmonellosis in foodborne illness-related deaths, and poses significant implications as a foodborne outbreak illness, particularly because of its high case fatality rates and severe effects on vulnerable populations such as pregnant women, newborns, older people and immunocompromised individuals.^50,51^ Foodborne botulism, while less prevalent compared with other foodborne illnesses, has also been associated with high case fatality rates in Africa. A notable instance in Ethiopia, where a family outbreak linked to traditional foods resulted in a staggering 50% case fatality rate, further emphasizes the lethal potential of this illness when it occurs.^13,21,23^

The current study revealed different categories of food products responsible as vehicles in microbial foodborne outbreaks, such as vegetables, fish, seafood, cereals, legumes, tuber and traditional African foods. However, processed and raw meats were the most implicated food vehicles in the microbial outbreaks in Africa. This predominance is strongly linked to the inherent vulnerability of meats to contamination during various stages, including slaughter, processing and storage, as reported by studies covered in this review. Many abattoirs in Africa regions lack essential facilities such as isolation pens and cooling equipment, leading to unhygienic meat handling. Inadequate handwashing, with 82.6% of workers untrained, contributes to poor hygiene practices, exacerbating the risk of contamination.^52–56^ The listeriosis outbreak in South Africa, which resulted in >1000 cases and >200 deaths, demonstrates the consequences of meat contamination. The high protein and moisture content of meat, combined with improper handling and storage, create an ideal environment for pathogens such as *S. enterica, L. monocytogenes* and *E. coli O157* to survive and grow, leading to widespread outbreaks and significant public health concerns.^57,58^

By contrast, other food categories, such as fish and seafood, were less frequently implicated. This may be due to lower consumption rates or the rapid spoilage of these foods, which discourages prolonged storage in settings with limited refrigeration.^59,60^

Traditional African and cultural foods, although nutritious, face safety challenges.^61^ Many are produced in informal settings under unhygienic conditions, leading to chemical and microbial contamination.^62^ For instance, *C. botulinum* outbreaks in Ethiopia were linked to consuming traditional foods such as kochi-kocha (cheese) and clarified butter. Similarly, *Cyanobacteria* (toxin) outbreaks in Comoros and Madagascar were associated with consuming sea turtles, a culturally valued food. Although less implicated, vegetables, salads and mayonnaise-based dishes present notable risks when consumed raw or inadequately washed, often becoming contaminated through non-compliance with hygiene standards and irrigation with polluted water or unhygienic handling. For instance, outbreaks linked to *V. cholerae* in raw vegetables and *S. aureus* in improperly handled salads highlight the vulnerability of fresh produce to contamination.^63,64^ Aside from practices that could lead to the contamination of fresh produce, their high moisture content may be conducive for the growth of both spoilage and pathogenic microbes.^65^

Food safety practices must be enhanced through comprehensive public awareness campaigns and increased financial investment in sanitation and food handling training to effectively control foodborne outbreaks in Africa. Priority should be given to controlling pathogenic microbes such as *Salmonella* spp., *S. aureus, L. monocytogenes, V. cholerae, Campylobacter* spp. and *E. coli*, which are prevalent in various food products. Implementing stricter hygiene standards and improving surveillance systems for foodborne illnesses, as well as ensuring the proper handling, storage and preparation of food, can significantly mitigate the risk of contamination. Additionally, investing in infrastructure, particularly in abattoirs and informal food production settings, is essential to ensure safer food supply chains. By addressing these key areas, public health can be significantly improved, reducing the burden of foodborne illnesses across the continent.

## Limitations

This systematic review focused on microbial foodborne outbreak incidents in Africa. Despite the valuable insights this study provides, it is subject to some limitations. First, the study may not capture the full extent of foodborne outbreaks in Africa due to the selection of articles, which could be at risk of bias due to the literature search and the inclusion criteria used.

Second, under-reporting of microbial foodborne outbreaks is particularly problematic in rural and underserved regions where surveillance systems and laboratory capacities are limited. While we attempted to mitigate this by searching multiple databases without location, language or year restrictions and including all study design types, the available literature likely under-represents outbreaks in less-resourced settings. Consequently, the findings may disproportionately reflect outbreaks from urban or better-resourced areas, and Africa's burden of foodborne illness may be underestimated. Future reviews should consider incorporating gray literature and local health authority records, as well as reaching out to non-governmental organizations to improve coverage. Strengthening surveillance infrastructure in rural areas is also critical to reducing this bias and ensuring more accurate estimates of the burden of African microbial foodborne disease.

Additionally, the absence of laboratory confirmation, the implicated foods and the microbial pathogen in some cases of the reported data reduces the certainty of this review. Furthermore, the diversity of food vehicles and pathogens complicates the development of targeted interventions. While some outbreaks are linked to specific foods, such as poultry meat or processed meats, others involve a wide range of foods, making it challenging to prioritize control measures. This heterogeneity reflects the complexity of African food systems, where informal markets and traditional practices play a significant role in food distribution and consumption.

These details are critical to developing future prevention and control measures.

## Conclusion

The current review highlights the significant public health burden posed by microbial foodborne outbreaks in Africa, driven by systemic challenges such as poor sanitation, inadequate food safety practices, insufficient infrastructure and limited public awareness. *Salmonella* spp., particularly *S. enterica*, emerges as the leading pathogen, causing most outbreaks across diverse food systems, including poultry, processed foods and beverages, with alarming prevalence and infection rates. *S. aureus* is also a major contributor due to its ability to produce heat-stable toxins, while *L. monocytogenes* and *C. botulinum*, although less common, are associated with high fatality rates, as seen in the devastating listeriosis outbreak in South Africa. The predominance of processed and raw meats as vehicles for contamination underscores the critical need for improved hygiene and infrastructure in meat production and handling, while traditional African foods and fresh produce also pose notable risks due to unhygienic conditions and improper handling. Addressing these challenges requires urgent investment in food safety infrastructure, improved hygiene practices, enhanced public awareness and stricter regulatory frameworks to mitigate the alarming health and socioeconomic impacts of foodborne illnesses across the continent.

## Supplementary Material

ihaf058_Supplemental_File

## Data Availability

All the supporting data are presented in the manuscript and supplementary files.
