# Supplementary material for: Microbial foodborne outbreaks in Africa: a systematic review
Source: Int Health. 2025 Jun 24;17(6):893–902. doi: 10.1093/inthealth/ihaf058 (PMC12585584; doi:10.1093/inthealth/ihaf058)
Supplement: ihaf058_Supplemental_File [file ihaf058_supplemental_file.docx]

**Supplementary Materials**

**Microbial Foodborne Outbreaks in Africa: A Systematic review.**

Famous K. Sosah^1^, and Eric S. Donkor^1^ **^*^**

^1^ Department of Medical Microbiology, University of Ghana Medical School, Accra, Ghana.

* Corresponding Author: **(Eric S Donkor)** Email: esampane-donkor@ug.edu.gh

**Supplementary Table 1- SEARCH STRING**

| DATABASE |  | SEARCH STRING | RESULT | DATE  TIME |
| --- | --- | --- | --- | --- |
| PUBMED |  |  | 473 | 5/10/24  09:32:12 |
|  |  | ("Foodborne Diseases"[Mesh] OR "foodborne outbreak*" OR "foodborne illness*" OR "foodborne infection*" OR "food poisoning" OR "food contamination" OR "gastroenteritis" OR "diarrhea" OR "enteritis") AND ("Infections"[Mesh] OR "microbial infection*" OR "bacterial infection*" OR "viral infection*" OR "parasitic infection*" OR "fungal infection*" OR "pathogen*" OR "Escherichia coli" OR "E. coli" OR "Salmonella" OR "Listeria" OR "Campylobacter" OR "Shigella" OR "Vibrio" OR "Clostridium" OR "Staphylococcus aureus" OR "Yersinia enterocolitica" OR "Bacillus cereus" OR "Brucella" OR "Norovirus" OR "Hepatitis A" OR "Rotavirus" OR "Astrovirus" OR "Sapovirus" OR "Adenovirus" OR "Cryptosporidium" OR "Giardia" OR "Entamoeba histolytica" OR "Cyclospora" OR "Toxoplasma" OR "Taenia" OR "Trichinella" OR "Ascaris" OR "Strongyloides" OR "Anisakis" OR "Candida" OR "Aspergillus" OR "Penicillium" OR "Fusarium") AND ("Africa"[Mesh] OR "Africa" OR "Sub-Saharan Africa" OR "North Africa" OR "West Africa" OR "East Africa" OR "Central Africa" OR "Southern Africa" OR "Algeria" OR "Angola" OR "Benin" OR "Botswana" OR "Burkina Faso" OR "Burundi" OR "Cabo Verde" OR "Cameroon" OR "Central African Republic" OR "Chad" OR "Comoros" OR "Congo" OR "Democratic Republic of the Congo" OR "Djibouti" OR "Egypt" OR "Equatorial Guinea" OR "Eritrea" OR "Eswatini" OR "Ethiopia" OR "Gabon" OR "Gambia" OR "Ghana" OR "Guinea" OR "Guinea-Bissau" OR "Ivory Coast" OR "Côte d'Ivoire" OR "Kenya" OR "Lesotho" OR "Liberia" OR "Libya" OR "Madagascar" OR "Malawi" OR "Mali" OR "Mauritania" OR "Mauritius" OR "Morocco" OR "Mozambique" OR "Namibia" OR "Niger" OR "Nigeria" OR "Rwanda" OR "São Tomé and Príncipe" OR "Senegal" OR "Seychelles" OR "Sierra Leone" OR "Somalia" OR "South Africa" OR "South Sudan" OR "Sudan" OR "Tanzania" OR "Togo" OR "Tunisia" OR "Uganda" OR "Zambia" OR "Zimbabwe") |  |  |
| SCOPUS |  |  | 497 | 5/10/24  14:07:08 |
|  |  | ("foodborne outbreak*" OR "foodborne illness*" OR "foodborne disease*" OR "foodborne infection*" OR "food poisoning" OR "food contamination" OR "gastroenteritis" OR "diarrhea" OR "diarrhoea" OR "enteritis") AND ("microbial infection" OR "bacterial infection" OR "viral infection" OR "parasitic infection" OR "fungal infection" OR "pathogen*" OR "Escherichia coli" OR "E. coli" OR "Salmonella" OR "Listeria" OR "Campylobacter" OR "Shigella" OR "Vibrio" OR "Clostridium" OR "Staphylococcus aureus" OR "Yersinia enterocolitica" OR "Bacillus cereus" OR "Brucella" OR "Norovirus" OR "Hepatitis A" OR "Rotavirus" OR "Astrovirus" OR "Sapovirus" OR "Adenovirus" OR "Cryptosporidium" OR "Giardia" OR "Entamoeba histolytica" OR "Cyclospora" OR "Toxoplasma" OR "Taenia" OR "Trichinella" OR "Ascaris" OR "Strongyloides" OR "Anisakis" OR "Candida" OR "Aspergillus" OR "Penicillium" OR "Fusarium") AND ("Africa" OR "Sub-Saharan Africa" OR "North Africa" OR "West Africa" OR "East Africa" OR "Central Africa" OR "Southern Africa" OR "Algeria" OR "Angola" OR "Benin" OR "Botswana" OR "Burkina Faso" OR "Burundi" OR "Cabo Verde" OR "Cameroon" OR "Central African Republic" OR "Chad" OR "Comoros" OR "Congo" OR "Democratic Republic of the Congo" OR "Djibouti" OR "Egypt" OR "Equatorial Guinea" OR "Eritrea" OR "Eswatini" OR "Ethiopia" OR "Gabon" OR "Gambia" OR "Ghana" OR "Guinea" OR "Guinea-Bissau" OR "Ivory Coast" OR "Côte d'Ivoire" OR "Kenya" OR "Lesotho" OR "Liberia" OR "Libya" OR "Madagascar" OR "Malawi" OR "Mali" OR "Mauritania" OR "Mauritius" OR "Morocco" OR "Mozambique" OR "Namibia" OR "Niger" OR "Nigeria" OR "Rwanda" OR "São Tomé and Príncipe" OR "Senegal" OR "Seychelles" OR "Sierra Leone" OR "Somalia" OR "South Africa" OR "South Sudan" OR "Sudan" OR "Tanzania" OR "Togo" OR "Tunisia" OR "Uganda" OR "Zambia" OR "Zimbabwe") |  |  |
| GOOGLE SCHOLAR |  | "foodborne outbreaks" OR "food-borne outbreaks" OR "foodborne illness" AND "microbial infection" OR "bacterial infection" OR "viral infection" OR "parasitic infection" AND "Africa" | 435 | 07/10/24  11:22:17 |
| WEB OF  SCIENCE |  | ("foodborne" OR "food-borne" OR "food associated") AND ("outbreak" OR "epidemic" OR "infection" OR "illness") AND ("microbial" OR "bacteria" OR "virus" OR "parasite" OR "fungi" OR "pathogen") AND ("Africa" OR "Sub-Saharan Africa" OR "North Africa" OR "West Africa" OR "East Africa" OR "Central Africa" OR "Southern Africa") | 146 | 07/10/24  15:32:01 |

Supplementary Table 2: PRISMA Checklist (Page *et al.*, 2021)

| **Section and Topic** | **Item #** | **Checklist item** | **Reported on page** |
| --- | --- | --- | --- |
| **TITLE** | | |  |
| Title | 1 | Identify the report as a systematic review. | 1 |
| **ABSTRACT** | | |  |
| Abstract | 2 | See the PRISMA 2020 for the Abstracts checklist. | 1 |
| **INTRODUCTION** | | |  |
| Rationale | 3 | Describe the rationale for the review in the context of existing knowledge. | 2 |
| Objectives | 4 | Provide an explicit statement of the objective(s) or question(s) the review addresses. | 2 |
| **METHODS** | | |  |
| Eligibility criteria | 5 | Specify the inclusion and exclusion criteria for the review | 2-3 |
| Information sources | 6 | Specify all databases, registers, websites, organizations, reference lists and other sources searched. Specify the date when each source was last searched or consulted. | 2-3 |
| Search strategy | 7 | Present the full search strategies for all databases, registers, and websites, including any filters and limits used. | 2-3 |
| Selection process | 8 | Specify the methods used to decide whether a study met the inclusion criteria of the review, including how many reviewers screened each record and each report retrieved, whether they worked independently, and if applicable, details of automation tools used in the process. | 3 |
| Data collection process | 9 | Specify the methods used to collect data from reports, including how many reviewers collected data from each report, and whether they worked independently. | 3-4 |
| Data items | 10a | List and define all outcomes for which data were sought. Specify whether all results that were compatible with each outcome domain in each study were sought (e.g. for all measures, time points, analyses), and if not, the methods used to decide which results to collect. | 3-4 |
|  | 10b | List and define all other variables for which data were sought (e.g. participant and intervention characteristics, funding sources). Describe any assumptions made about any missing or unclear information. | Not applicable |
| Study risk of bias assessment | 11 | Specify the methods used to assess the risk of bias in the included studies, including details of the tool(s) used, how many reviewers assessed each study and whether they worked independently, and if applicable, details of automation tools used in the process. | Not Applicable |
| Effect measures | 12 | Specify for each outcome the effect measure(s) (e.g. risk ratio, mean difference) used in the synthesis or presentation of results. | Not applicable |
| Synthesis methods | 13a | Describe the processes used to decide which studies were eligible for each synthesis (e.g. tabulating the study intervention characteristics and comparing against the planned groups for each synthesis (item #5)). | 3 |
|  | 13b | Describe any methods required to prepare the data for presentation or synthesis, such as handling of missing summary statistics, or data conversions. | Not applicable |
|  | 13c | Describe any methods used to tabulate or visually display the results of individual studies and syntheses. | 3-4 |
|  | 13d | Describe any methods used to synthesize results and provide a rationale for the choice(s). If meta-analysis was performed, describe the model(s), method(s) to identify the presence and extent of statistical heterogeneity, and software package(s) used. | 3-4 |
|  | 13e | Describe any methods used to explore possible causes of heterogeneity among study results (e.g. subgroup analysis, meta-regression). | Not applicable |
|  | 13f | Describe any sensitivity analyses conducted to assess the robustness of the synthesized results. | 4 |
| Reporting bias assessment | 14 | Describe any methods used to assess the risk of bias due to missing results in a synthesis (arising from reporting biases). | Not applicable |
| Certainty assessment | 15 | Describe any methods used to assess certainty (or confidence) in the body of evidence for an outcome. | Not applicable |
| **RESULTS** | | |  |
| Study selection | 16a | Describe the results of the search and selection process, from the number of records identified in the search to the number of studies included in the review, ideally using a flow diagram. | 4 |
|  | 16b | Cite studies that might appear to meet the inclusion criteria, but which were excluded, and explain why they were excluded. | Not applicable |
| Study characteristics | 17 | Cite each included study and present its characteristics. | 9 |
| Risk of Bias in Studies | 18 | Present assessments of risk of bias for each included study. | Not Applicable |
| Results of individual studies | 19 | For all outcomes, present, for each study: (a) summary statistics for each group (where appropriate) and (b) an effect estimate and it's precision (e.g. confidence/credible interval), ideally using structured tables or plots. | Not applicable |
| Results of syntheses | 20a | For each synthesis, briefly summarise the characteristics and risk of bias among contributing studies. | Not applicable |
|  | 20b | Present results of all statistical syntheses conducted. If meta-analysis was done, present for each the summary estimate and its precision (e.g. confidence/credible interval) and measures of statistical heterogeneity. If comparing groups, describe the direction of the effect. | Not applicable |
|  | 20c | Present results of all investigations of possible causes of heterogeneity among study results. | Not applicable |
|  | 20d | Present results of all sensitivity analyses conducted to assess the robustness of the synthesized results. | Not applicable |
| Reporting biases | 21 | Present assessments of risk of bias due to missing results (arising from reporting biases) for each synthesis assessed. | Not applicable |
| Certainty of evidence | 22 | Present assessments of certainty (or confidence) in the body of evidence for each outcome assessed. | Not applicable |
| **DISCUSSION** | | |  |
| Discussion | 23a | Provide a general interpretation of the results in the context of other evidence. | 12-15 |
|  | 23b | Discuss any limitations of the evidence included in the review. | 14 |
|  | 23c | Discuss any limitations of the review processes used. | 14 |
|  | 23d | Discuss the implications of the results for practice, policy, and future research. | 14 |
| **OTHER INFORMATION** | | |  |
| Registration and protocol | 24a | Provide registration information for the review, including the register name and registration number, or state that the review was not registered. | Not applicable |
|  | 24b | Indicate where the review protocol can be accessed, or state that a protocol was not prepared. | Not applicable |
|  | 24c | Describe and explain any amendments to the information provided at registration or in the protocol. | Not applicable |
| Support | 25 | Describe sources of financial or non-financial support for the review, and the role of the funders or sponsors in the review. | 15 |
| Competing interests | 26 | Declare any competing interests of review authors. | 15 |
| Availability of data, code, and other materials | 27 | A report of which of the following are publicly available and where they can be found: template data collection forms; data extracted from included studies; data used for all analyses; analytic code; any other materials used in the review. | Not applicable |

Reference

Page, M.J. *et al.* (2021) ‘The PRISMA 2020 statement: An updated guideline for reporting systematic reviews’, *PLOS Medicine*, 18(3), p. e1003583. Available at: https://doi.org/10.1371/journal.pmed.1003583.
